# Improving Primary Care Quality Through Supportive Supervision and Mentoring: Lessons From the African Health Initiative in Ethiopia, Ghana, and Mozambique

**DOI:** 10.9745/GHSP-D-21-00667

**Published:** 2022-09-15

**Authors:** African Health Initiative Partnership Collaborative for Supportive Supervision and Mentoring

**Affiliations:** a Members listed at the end of the article.

## Abstract

**Introduction:**

Supportive supervision and mentoring (SSM) is crucial to primary care quality and effectiveness. Yet, there is little clarity on how to design and implement SSM and make it sustainable in primary health care (PHC) systems. The 3 African Health Initiative partnership projects introduced strategies to do this in Ethiopia, Ghana, and Mozambique. We describe: (1) how each partnership adapted SSM implementation strategies, (2) the dynamics of implementation and change that ensued after intervening within PHC systems, and (3) insights on the SSM sustainability as a mainstay of PHC.

**Methods:**

Researchers from each project collaboratively wrote a cross-country protocol based on those objectives. For this, they adapted implementation science frameworks—the Exploration, Preparation, Implementation, and Sustainment model and the Consolidated Framework for Implementation Research—through a qualitative theme reduction process. This resulted in harmonized lines of inquiry on the design, implementation, and potential sustainability of each project’s SSM strategy. In-depth interviews and focus group discussions were conducted with stakeholders from PHC systems in each country and thematic analyses ensued.

**Results:**

Across the projects, SSM strategies acquired multiple components to address individual, systems, and process-related determinants. Benefits arose from efforts that addressed worker-level attitudes and barriers, promoted a wider learning environment, and enhanced collaborative structures and tools for monitoring performance. Peer exchanges and embedded implementation research were critical to the perceived effectiveness of SSM strategies.

**Discussion:**

Despite differences in their approach to SSM implementation, there are common crucial ingredients across the SSM strategies of the 3 AHI partner projects from which important lessons arise: (1) positioning learning and adaptation opportunities within the routine workings of PHC systems, facilitation, and technical support to reflect and utilize new knowledge; (2) multisectoral collaboration, particularly with academic organizations; and (3) building PHC decision-makers’ and implementation teams’ capacity for evidence-informed change.

## Abstract (precis)

Systematic approaches to positioning technical support, enhancing systems, and promoting sustainment are crucial to strengthening supportive supervision and mentoring in primary health care systems. The African Health Initiative projects in Ethiopia, Ghana, and Mozambique have lessons to share from such experiences that stakeholders can apply to similar efforts in other countries.

## Teaser

**Key Findings:**

- Benefits arose from supportive supervision and mentoring strategies that addressed multilevel factors surrounding the point of care. These factors include worker-level attitudes and individual barriers to performance improvement, the extent to which learning and adapting are accepted and encouraged in the wider environment, and district-level collaborative structures and resources available to managers and teams for monitoring performance.
- Peer exchanges between primary health care (PHC) teams, including embedded implementation researchers, were instrumental to the success of supportive supervision and mentoring.

**Key Implications:**

- Stakeholders that have influence over the practice, processes and context of supportive supervision and mentoring in primary care settings must be engaged in diagnosing quality problems and using data to cocreate solutions that address the factors that affect quality.
- Managers and implementing partners may consider ways to incorporate learning and collaboration between PHC teams across jurisdictions and foster multisectoral partnerships to sustain motivation and build capacity for better supportive supervision, mentoring, and performance improvement.

Resumo em português no final do artigo.

## INTRODUCTION

The World Health Organization (WHO) *World Health Report 2008* and subsequent conferences that culminated with the Declaration of Astana in 2018 revitalized the global movement on primary health care (PHC). Formally launched 40 years earlier, PHC is a holistic strategy to prevent the fragmentation of health care into discrete initiatives, promote the preventive aspects of public health, address underlying structural inequalities, and address community health needs at health systems’ peripheries.^1^^–^^4^ Strategies for achieving universal coverage of PHC have focused on the removal of financial barriers to care, social determinants of health, workforce interventions to staff remote facilities and communities, and innovative technologies appropriate to PHC settings.^5^^–^^9^ Implementation experience relates that these efforts will not come to fruition in the absence of concomitant efforts to ensure the quality of PHC and health care worker performance.^10^ Indeed, there are numerous examples of gaps in the extension and maintenance of high-quality care in clinical and community PHC environments.^11^^,^^12^

Currently, the evidence on supportive supervision, collaborative mentoring, and coaching to improve PHC is mixed. A systematic review by Bosch-Capblanch et al. on the effect of managerial supervision to improve PHC in low- and middle-income countries (LMICs) concluded that it was uncertain whether supervision has a substantive, positive, long-term effect.^13^ A review by Bailey et al. on the effect of supportive supervision to improve PHC in sub-Saharan Africa reported that supportive supervision was clearly associated with improved health worker job satisfaction and motivation, whereas its relationship with better clinical competence was mixed.^14^ However, studies have shown that applying such strategies with a focus on specific clinical issues within PHC, such as integrated maternal and child health services, mental health care, testing and treatment for HIV and other chronic diseases, has led to improved service coverage and utilization, provider performance and quality, and patient-level health and quality of life outcomes.^15^^–^^17^ Elsewhere in the literature authors note the absence of practical support aimed at helping to instantiate and build capacity to sustain effective supportive supervision, collaborative mentoring, and coaching in the wider context of frontline PHC provision in LMICs. For example, recent review of the literature on this topic found that there was little clarity on what defines supervision and mentoring, the design and implementation of these interventions, and how to incorporate them into health systems.^18^

This article describes the experience of 3 PHC systems-implementation science partnerships in Ethiopia, Ghana, and Mozambique that conducted supportive supervision and mentoring (SSM) interventions to promote the quality of PHC. It draws upon these projects’ rigorous documentation of implementer and stakeholder perceptions of SSM implementation. Although the cultural and health systems contexts differed across the partnerships, similar theoretical and action frameworks for planning implementation and embedding research were used by each team. This article uses findings from the midline process evaluation of each project. In doing so, it describes: (1) how each partnership adapted SSM implementation strategies and optimized them for success, (2) the dynamics of implementation and change that ensued from operationalizing SSM within PHC systems, and (3) insights on the sustainability of SSM as a mainstay of PHC. Recommendations for applying SSM in pursuit of universal coverage of PHC are reviewed and discussed.

We describe the experience of 3 PHC systems-implementation science partnerships in Ethiopia, Ghana, and Mozambique that conducted SSM interventions to promote the quality of PHC.

## THE AFRICAN HEALTH INITIATIVE IN ETHIOPIA, GHANA, AND MOZAMBIQUE

Since 2009, the African Health Initiative (AHI) of the Doris Duke Charitable Foundation (DDCF) has supported collaborations between ministries of health (MOHs), PHC implementation leaders, and academic groups to embed implementation research in PHC systems in 6 countries. The first phase of the AHI, 2009–2014, demonstrated the proof of concept that locally adapted PHC implementation strategies could effectively accelerate universal health care achievement in Ghana, Mozambique, Rwanda, Tanzania, and Zambia. The second phase, which started in 2015, shifted the focus to replication and supporting the scale-up of effective strategies in 3 countries, Ghana, Mozambique, and Ethiopia, whose partnership joined the AHI at that time. During phase 2, partnerships in Ethiopia, Ghana, and Mozambique developed and carried out context-specific strategies to improve PHC quality.

In Ethiopia, the Data Use Partnership of John Snow International, Addis Ababa University, municipal health authorities, and the MOH have worked in 3 subcities of Addis Ababa and blended efforts to improve frontline health care worker performance by strengthening their use of data for decision making.

In Ghana, the CHPS+ Project (named after the country’s PHC strategy, the Community-based Health Planning and Services Program) of the Regional Institute for Population Studies, University for Development Studies, University of Health and Allied Sciences, Columbia University Mailman School of Public Health, and the Ghana Health Services (GHS) works at multiple levels of care to extend facilitative supervision (FSV) to the community level. CHPS+ provides logistics support and catalytic funds to subdistricts to carry out FSV to community health workers and holds peer-learning exchanges between districts.

In Mozambique, Health Alliance International, the University of Washington, and the MOH carried out an iterative 3-step process of data-driven SSM in 12 districts. This proceeds cyclically, by way of (1) data quality audits and strengthening, (2) blended district- and facility-level audit and feedback sessions and action planning, and (3) targeted SSM and financial support to implement action plans.

## METHODS

### The AHI SSM Working Group

The AHI SSM Working Group, which includes representatives from each project, a working group lead, and a coordinator, conducted in-person and remote exchanges between May and December 2019. These exchanges resulted in 3 project-specific protocols describing small studies on SSM that teams would conduct in each country and an overarching concept paper that specified the common lines of inquiry that each study would pursue. Between January and June 2020, AHI partnerships fielded qualitative primary data collection, including in-depth interviews (IDIs) and focus group discussions (FGDs). Participants were recruited if they had at least 2 years of continuous experience as implementation-level stakeholders of each of the projects, including direct involvement in their respective SSM components.

In Ethiopia, data were collected in 3 subcities of Addis Ababa (Yeka, Akaki-Kaliti, and Ledeta). FGDs included between 6 and 10 participants. All data collection was conducted and transcribed in Amharic. Digital recordings of interviews were translated by interviewers into English. The average IDI was 30 minutes and the average FGD was 2 hours in duration.

In Ghana, the study was carried out in 4 districts in the Northern Region (Gusheigu and Kumbungu) and Volta Region (Central Tongu and Nkwanta South). FGD included 8–12 participants. Both IDIs and FGDs were conducted in English, digitally recorded, and then transcribed by interviewers. The average IDI lasted 35 minutes and the average FGD lasted 90 minutes.

In Mozambique, data were collected in 4 districts, Chimoio and Gondola (Manica Province) and Beira and Dondo (Sofala Province). FGDs had 6–12 participants. All data collection was conducted, transcribed, and analyzed in Portuguese. The final report from the Mozambique team was written in English by senior-level researchers on the project. The average IDI was 45 minutes and the average FGD lasted 90 minutes.

In all 3 projects, data collectors had relevant experience conducting data collection on health systems issues. They either had first-level university degrees in social and/or health sciences and received a week of training on the study protocol.

Researchers in Ghana and Mozambique benefited from the availability of qualitative process evaluation data that had been collected earlier in their project cycles on the same themes and with the same participants. Accordingly, they chose to include these transcripts in the analysis. Researchers from Ethiopia only used the qualitative data collected for this working group; however, they benefited from rich, firsthand knowledge of implementation processes and context of project team members that helped co-author their report. Table 1 describes all data used for the analysis reported in this article.

**TABLE 1.** Data Collection and Sampling for Each African Health Initiative Project

	**In-Depth Interviews**		**Focus Group Discussions**	
**Country**	**No.**	**Participants**	**No.**	**Participants**
Ethiopia	40	Regional and woreda-level (i.e., district) health management team; health center staff (head, deputy head, health information technician, and maternal/child health care workers)	6	Regional and woreda-level health management team; health center staff (head, deputy head, health information technician, and maternal/child health care workers)
Ghana	34	District health management team and community health officers	52	Community members, community health volunteers, subdistrict health team
Mozambique	7	District health managers	39	Frontline nurses

### Analytic Approach

Scientific staff of the projects in Ethiopia, Ghana, and Mozambique carried out the analysis and wrote project-specific reports. These staff come from their respective countries and were all trained to graduate level in social and or public health sciences and had experience performing qualitative data analysis independently using the approaches described below. In each country, these researchers carried out the qualitative analysis that followed a grounded theory approach. First, they employed “constant comparison,” (i.e., deeply examining transcripts, and comparing interpretations of text with existing findings as they emerge, thereby deriving themes to explore during analysis, and sets of codes for each theme).^19^ Then, analysts carried out a framework analysis and reorganized data into coded segments that related to the themes.^20^ This resulted in a report from each country. As per the working group’s overarching concept paper, each report contained 4 sections: (1) the development of SSM strategies, (2) how projects operationalized their strategies in local health systems, (3) barriers and facilitators to effective implementation, and (4) programming recommendations and lessons learned. Working group members sent their reports to the working group lead and coordinator who conducted a thematic analysis of each report and developed a codebook.^21^ To develop the codes, the working group coordinator adapted constructs supplied by 3 implementation science frameworks: Lewis et al.’s framework for understanding causal pathway models (CPM) in implementation science, the exploration, preparation, implementation, sustainment (EPIS) framework for facilitating evidence-based practices in health systems, and^22^^–^^24^ the Consolidated Framework of Implementation Research (CFIR), which helps to illuminate factors that influence implementation effectiveness.^25^^,^^26^ The coordinator coded the 3 reports and used memos to document thematic insight using qualitative software, QSR-Nvivo (version 11). Themes were based on further analysis of coded segments of the reports, including quotations obtained from IDI participants. This was carried out in Microsoft Word. The results were confirmed by working group members who then collaborated on interpreting the results and selecting programming recommendations. The Figure illustrates how implementation science frameworks and qualitative data were triangulated for this comparative analysis.

**FIGURE.**
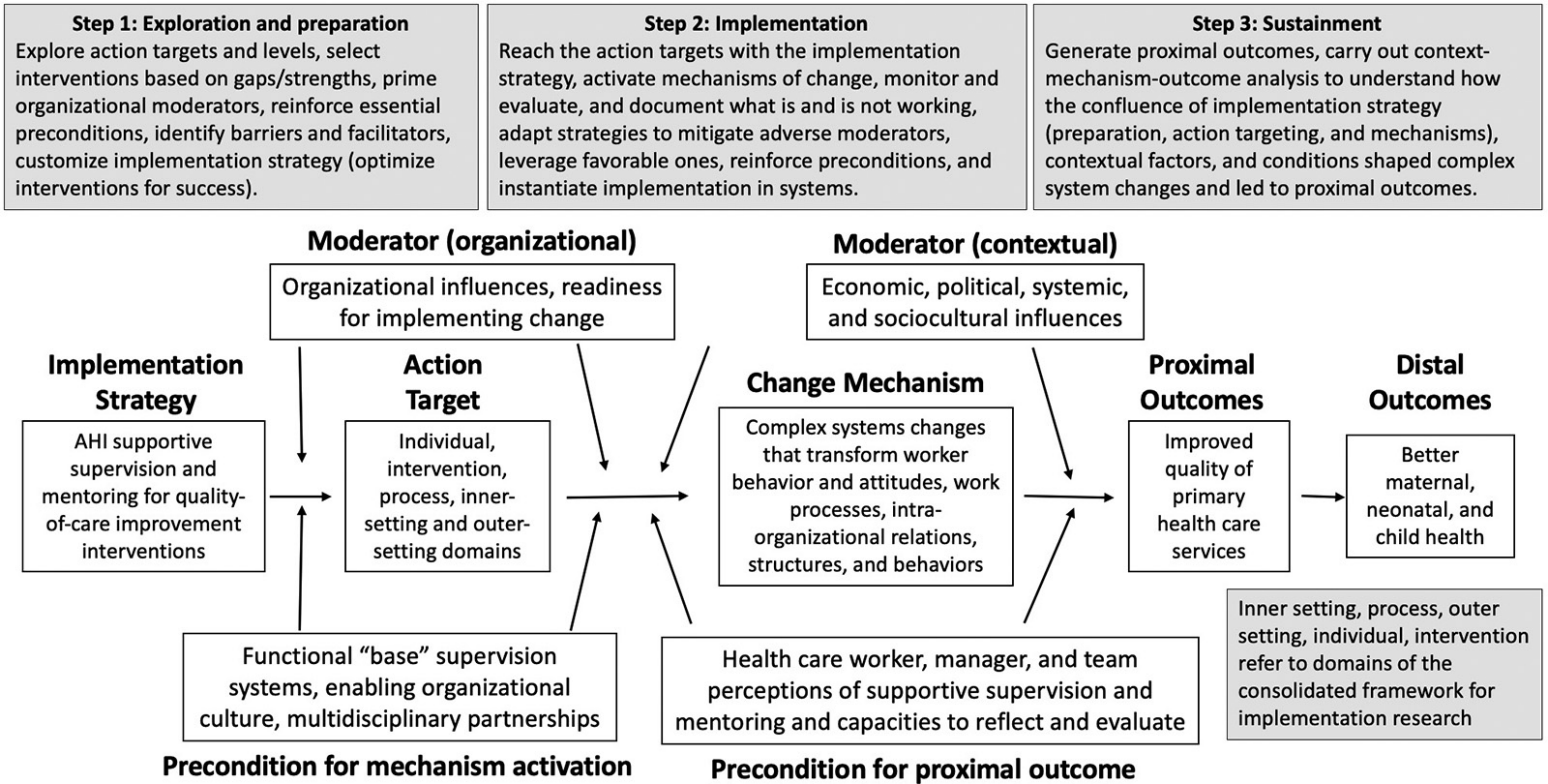
Implementation Science Frameworks and Qualitative Data Were Triangulated for This Comparative Analysis Across 3 Countries Abbreviation: SSM, supportive supervision and mentoring.

The projects started with an **exploratory and preparatory phase**.^23^ During this first phase, teams sought to understand the challenges that characterize PHC systems and root causes that underlie suboptimal performance, pinpoint factors to address through interventions, and thereby specify their SSM implementation strategies. Subsequently, the projects **instantiated SSM implementation into health systems**, linking interventions with the “action targets” where barriers and facilitators to PHC performance operate and trigger the change mechanism hypothesized to produce intended outcomes. During this second phase, teams identified intervening factors and processes that mediate the relationship between the strategy and outcomes and optimized implementation accordingly. In the third, and current, phase of implementation, the AHI projects **promote sustainment** of the “proximal outcomes.” To do so, teams articulate contextual factors that moderate implementation effectiveness and take actions to harness or mitigate these, often by bolstering critical preconditions. The Supplement Table specifies the CPM for the 3 SSM interventions.

### Ethical Considerations

All study participants were informed of their rights to confidentiality and privacy and to withdraw from the study at any time, the risks and benefits of participating in data collection, and measures the study would take to ensure that they would be treated with respect. After each participant provided their informed consent to participate, they signed an informed consent form. Institutional Review Boards (IRB) in each country reviewed these procedures and the data collection materials and granted each project ethical clearance and permission to conduct this research.

## RESULTS

Results comprise synthesis and interpretation of findings reported by the AHI teams, including, where possible, direct quotations of stakeholders.

### Phase 1: Understanding Gaps, Adapting, and Specifying SSM Implementation Strategies

The initial steps each AHI partnership took aimed at obtaining insight on the determinants of frontline worker performance and ways that an SSM intervention can address them. During the first phase of exploration and preparation, AHI teams identified factors at the process, individual, outer setting, and inner setting levels (Table 2). For example, the Data Use Partnership in Ethiopia convened a consultative workshop with national-level stakeholders from the MOH, academia, and civil society to understand the barriers to improving supervision systems to reach *woreda* (district) level health care workers. This drew attention to the lack of procedural guidance and structures for engaging mentors, supervisors, and health care workers in the use of data at the point of care. The AHI team brought these notions to woredas to learn about these gaps at the management and facility levels and what support might help. This revealed how supervision and accountability mechanisms had atrophied in the absence of appropriate tools and skills for circulating feedback between frontline and supervision levels.

**TABLE 2.** Determinants, Where They Operate, and the Strategies to Address Them During the Exploration and Preparation Phase in the AHI Partnership Projects

	**Ethiopia**	**Ghana**	**Mozambique**
**Determinants:** The barriers to high-quality PHC that each project sought to address			
Individual level	Poor use of data at the point of care	Supervisors’ beliefs about what supervision is and their roles as supervisor vis-à-vis the frontline worker	Poor use of data for management and point-of-care decision making
Process level	Absence of procedural guidance and planning on how to embed SSM	Lack of programmatic structure and technical guidance for supervision at subdistrict and CHPS levels	Weak institutional planning and coordination for supervision and frontline worker management
Inner setting	Unavailability of human resources for mentoring and poor supervision information flow and feedback loops	Inadequate resources and incentives for supportive supervision to frontline workers	Inadequate human and financial resources for SSM
Outer setting	Inadequate structures for mentorship in the national HIS program		Absence of incentives to motivate performance improvement
**Implementation strategy:** The intervention that each team adapted according to the context and barriers to high-quality PHC			
	CBMP	FSV	SSM
	Adaptation of MOH HIS mentorship guide	District-to-district peer learning exchanges aimed at supervisor capacity building, experience sharing	Building frontline worker and PHC team capacity to record and manage high-quality service statistics and use for point-of-care decision making and benchmarking.
	Multisectoral partnerships for mentorship selection and training	Strengthened tools and procedural guidance for carrying out routine FSV	Facilitation of planning and engagement of frontline worker teams within/across districts through a district-level audit and feedback intervention
	Health worker training in data use for quality improvement	Organizational incentives to support and motivate ongoing conduct of FSV of frontline workers at subdistrict and CHPS level	Transparent data-driven prioritization and customization of SSM to PHC teams and promotion of patients’ needs during SSM
	Standardization of follow up/coaching/ mentoring practices and feedback loops linking management to point of care		Incentives to motivate PHC team action planning and execution

Abbreviations: AHI, African Health Initiative; CBMP, capacity-building and mentorship program; CHPS, Community-based Health Planning and Services Program; FSV, facilitative supervision visits; HIS, health information system; MOH, Ministry of Health; PHC, primary health care; SSM, supportive supervision and mentoring.

During the first phase, each AHI partnership sought insight on the determinants of health care worker performance and ways that an SSM intervention could address them.

In Ghana, the exploration phase started with qualitative health systems appraisals (QSA) of stakeholder perspectives on CHPS’ functioning. They were conducted at the district, subdistrict, and CHPS zone level by way of IDI and FGD that probed for perceptions of managers and frontline workers and their supervision experiences. An early QSA round indicated that district-level support and linkages to the point of care waned as PHC coverage expands. Supervisors pointed out insufficient funds, transport, and logistics for outreach to CHPS zones and revealed a tendency of supervisors to neglect performance improvement and emphasize data validation instead. Frontline workers lamented the prominence of faultfinding and the dearth of constructive support they receive from supervisors.

*This is a human institution where we have our strengths and weakness, but when they come, some of the supervisors dwell on our weaknesses instead of appreciating what you have been able to accomplish. At least [they should] make you understand that you could have done something this way or that way. Later you will come to the district for logistics to fill the gaps you have but you wouldn’t get it. …It is the gaps [supervisors] dwell on … and do not help you solve those problems.* —Community health officer, Ghana

In Mozambique, this first phase explored the context of weak institutional planning and coordination between district-level managers and frontline workers and how that affects the quality of care. It concluded that supervision combined with mentoring, coaching, and using accurate data will promote quality by informing the basis for customized in-service training. To mold that vision into strategy, Health Alliance International obtained input from project staff, district managers and supervisors, and frontline workers on the current SSM model. Informants reported that SSM diverts frontline worker attention from patient care and described poor data management and use at the point of care and in planning settings. District supervision capacity and resources for logistics were outstripped by the number of PHC sites that required SSM, which promoted a tendency to reduce the practice to an administrative follow-up.

*We [should] prioritize health facilities that are assumed to have more problems… evaluate indicators and try and see what facilities underperform compared to others; so, we prioritize those. We [should] go to those facilities to provide supervision and technical support. —*District manager of health services, Mozambique

With this, the Mozambique team identified the need for public benchmark reporting of PHC quality. This promotes friendly competition between sites and informs the basis for rationalizing the deployment of SSM to facilities that demonstrate the greatest need. Under this arrangement, SSM takes on administrative significance and engages clinical staff in identifying bottlenecks and problems and customizing solutions to them.

Thus informed, AHI partnerships in Ethiopia, Ghana, and Mozambique sought to devise implementation SSM strategies. They did so by holding a series of workshops and small collaborative sessions with policy decision makers involved as co-investigators of the respective projects, local health system implementation leaders (e.g., district and municipality), and academic partners from local universities. During these sessions, they reflected on the previously described findings, identified root causes of challenges (i.e., determinants), and prioritized courses of action that could trigger desired changes. All partnerships emphasized motivating and building capacity to plan, coordinate, and engage a wide range of stakeholders and adapting SSM practices for uptake in district-level management and point-of-care settings. In Ethiopia and Mozambique, teams also aimed at strengthening policy guidance on mentoring in the health information system (HIS) (Ethiopia) and public benchmark reporting of quality improvement plans at the district level (Mozambique) (Table 2).

### Phase 2: Introducing and Integrating SSM Implementation Strategies

During the second phase, the AHI partnerships instantiated implementation into health systems. They did so, first, by introducing their SSM strategies, integrating them into routine delivery systems, and directing them at the action targets identified in phase 1. Then, the teams monitored whether executing their strategies triggered change mechanisms that led to intended outcomes and the mediators through which that happened. In Ethiopia, Addis Ababa University (AAU) and City Administration Health Bureau collaborated to embed a modified mentorship approach in the national HIS. To ensure that mentorship had a permanent structure within the health system, the partners appointed subcity staff to mentor PHC at the woreda level and introduced teams of mentors of mentors. These teams comprised of AAU and the Regional Health Bureau staff who received training from AAU to master the use of the district health information system (DHIS-II), data quality audits, data visualization, and mentorship techniques. AAU and Regional Health Bureau counterparts then adapted these trainings into regimens they could cascade to their mentees, subcity supervisors whose job was to manage the performance of frontline workers at health posts in communities. New mentors of mentors responded favorably to the strategy.

*It helped us to understand about mentorship, what it is about which we had no idea before. If they had not provided us the training, we wouldn’t have had this improvement. We, mentors also had skill gaps on DHIS-2 which we raised at the meeting with AAU and received a 5-day training. The training helped us to become good mentors in our third round mentoring, where we provided support to health centers focusing on how to use DHIS-2*. —Health information mentor, Ethiopia

The teams monitored whether executing their strategies triggered change mechanisms that led to intended outcomes.

Mentors also attested to how the implementation strategy strengthened their ability to engage and plan with counterparts and promote team-based learning and growth.

*Trainings are more of interactive and following adult learning principles. For DHIS2 training, we use computers, and it is a practical training, not theoretically based. Other trainings are also facilitated in more interactive and participatory ways.* —Health information mentor, Ethiopia

*To mentor someone, you have to have better skills about the subject matter and formats which are used to record or report. When I say this, I don’t mean mentors must be trained in every aspect, but it is good to have comprehensive skills across services a mentor is supporting. If we don’t transfer skills on some subject matter in better ways, mentees don’t perceive us as problem solvers.* —FGD participant, Ethiopia

Stakeholders remarked on how SSM implementation changed the networks and communications of the municipal health system by connecting management with frontline workers through supervision and feedback loops.

*What I saw as good experience in this mentoring is that mentoring feedback is sent. These feedbacks contain strengths and gaps in detail which again I appreciate. We distribute the feedback to each concerned department so that they can take action to fill identified gaps.* —Health facility project coordinator, Ethiopia

In Ghana, the strategy to improve FSV occurred at regional, district, subdistrict, and community levels. A central element involved peer-learning visits between health management teams from CHPS+ districts to sites in the Upper East Region where the program originated. Exchanges oriented visiting teams to effective management practices that helped take CHPS to scale years before. These emphasized the development of a new tier of subdistrict supervisors, closer to the frontline, which became responsible for facilitating and monitoring the deployment of community health workers. In turn, district health management teams could address the more manageable tasks of directing subdistrict supervisors. Feedback from peer-learning visitors sums up their lessons learned.

*The other thing also was a strong subdistrict team; so, in fact when we came back immediately from the peer exchange in Upper East, we engaged our stakeholders and then formed a subdistrict team. But when we strengthened the subdistrict and then empowered them to go to the CHPS zones to conduct a facilitative support visit, it took that burden, kind of, from us. That’s a key thing we learned from the Upper East.* —District health management team member, Ghana

Peer exchanges also imparted a clear sense of how to adapt district support to frontline workers in CHPS+ areas.

*One other thing we learnt when we went there was monthly supervision, they were doing it consistently, we were doing it once in a while or none in the whole year. When we went there, they introduce us to the monitoring tools …that aid them to give feedback to the facility on areas that they were not doing well for them to strengthen.* —District health information officer, Ghana

*They brought us the FSV, which is now helping you to at least know the targeted places that you need to supervise on. You would not just go and say the things off head. You know what you are going to look at and what you are going to look for*. —Subdistrict health team member, Ghana

In addition, CHPS+ promoted a comprehensive understanding of individuals’ roles as mentors, which tended to be conducive to quality improvement.

*You coach them, the good things you recommend, the good things, and then you coach them, you put them in line with what they are not doing very well. You coach them as to how they will do it.* —District health management team member, Ghana

The partnership in Mozambique emphasized health care worker capacity building in data quality and use, making adaptations to district protocols for PHC action planning and coordinating SSM, and fostering better engagement between facility teams within districts. Frontline workers and teams, that are increasingly able to produce high-quality reports, engaged with counterparts from other facilities in their district during quarterly meetings called *reuniao de avaliacao dos dados* (district performance review and enhancement meetings). There, they shared progress and exchanged feedback on ways to improve the quality of care, which, in turn, informed facility teams’ future benchmarks and action plans.

*Data review helps very much to see if we are working or not and presentations help to share experiences. [District performance review and enhancement meetings] help develop action plans to address shortcomings. The plans are posted at a visible location in the health facility.* —Facility nurse, Mozambique

*There is competition among the health facilities, which contributes to improving data quality. These meetings are also a reminder to the districts about what is going on and what needs to be done.* —District manager, Mozambique

Based on this, district-level teams prioritized where to carry out SSM visits to audit action plans and which sites to designate as centers of excellence based on their record of performance improvement. These centers of excellence, in turn, would serve as sites for teams to visit for capacity-building purposes and for ways to foster a richer learning environment in districts. District supervisors felt that data-driven prioritization enables them to hold more focused and in-depth consultations.

*Based on the facility performance, we rank facilities as better or worse in meeting indicators [targets]. Then, 3 facilities are selected for technical support. Right? After discussion [during A&F meetings], we select 2 low performing and one high performing. We go to those facilities to provide supervision and technical support.* —District manager, Mozambique

Frontline workers that received SSM visits following the district performance review and enhancement sessions noted that the support they received from district supervisors contributed to improvement in their interpersonal skills.

*When they get to the health facilities, they are very caring. We feel at ease to relay our doubts and questions.* —Facility nurse, Mozambique

*During patients’ appointments, the interaction between the nurse and the patient has improved …. we now know how to listen to the patient, clarify doubts, and always explain the recommendations. This part nurses use to miss.* —Facility nurse, Mozambique

Stakeholders in Mozambique noted that the project deepened organizational consensus on the importance of data-driven decision making, audit, and feedback. Furthermore, they report that the SSM strategy was compatible with preexisting facets of district health systems and generated a greater impetus for strategic change, a better learning climate, and new opportunities for setting goals and giving feedback. One stakeholder described how provincial authorities, so influenced, took steps to facilitate learning and spread of the SSM model to non-AHI districts.

*Additionally, the province innovated, by including in the supervision teams, nurses from other districts with the argument of sharing experience*. —MOH program manager, Mozambique

Midpoint findings from all 3 projects were promising. The team in Ethiopia emphasized how their approach fostered administrative ownership of data use for decision making and quality improvement.

*Moreover, sense of ownership, especially on data quality and utilization, is created. Even mentorship is no longer conducted, making efforts to ensure use of quality data have become a habit nowadays. The performance monitoring team was also strong enough to take over the activities and use data as per the standard. They are doing these activities for their own sake.* —Regional monitoring and evaluation officer, Ethiopia

Midline QSA in Ghana revealed that their strategy has led to more effective FSV in CHPS zones.

*They are really playing their roles because when they come sometimes, they evaluate the activities we are going to do. They go through our registers, ONC, CWC, FP, and OPD registers and wherever we need improvement they make evaluations and show us the way forward because they don’t want to come and meet the same situation.* —Community health officer, Ghana

Furthermore, the partnership in Ghana fostered a commitment to FSV and teamwork across levels of care.

*In case they have any issue in terms of service delivery at the various facilities, they invite the subdistrict leader and then we also collaborate with the District Health Administration to see how the issue can be solved, and then in terms of service delivery, we are also responsible for providing them with drugs. We make sure that their work goes on smoothly, vaccines and any other form of logistics that they need at the various CHPS compounds.* —Subdistrict health team member, Ghana

Mid-project findings reported from Mozambique indicate that the SSM approach has been implemented with notable fidelity to the implementation strategy. By this time, all intervention districts had adopted the SSM model with no reported lapses in implementation. Of the 380 supervision visits, 265 (70%) had been prioritized as per deliberations during the district performance review and enhancement meetings. This translates into approximately 3 supervisions per prioritized facility during which district supervisors audited action plan achievement and gave feedback to frontline worker teams of 97 facilities (63% of facilities in the intervention districts) (Table 3).

**TABLE 3.** Results of Phase 2, Implementation of AHI Partnerships’ SSM Interventions in Ethiopia, Ghana, and Mozambique

	Ethiopia: CBMP	Ghana: CHPS+ FSV	Mozambique: SSM
Mediator: The processes through which the SSM strategies catalyzed changes			
Individual	Build frontline workers’ capacity in data management, use, and visualization	Increase supervisors’ confidence and self-efficacy vis-à-vis SSM for a clearer and more enthusiastic understanding of their role	Build self-efficacy of mentors and supportive relationship with mentees; improve knowledge and attitudes toward the intervention; improve data use skills
Process	Conduct cascade trainings to expand coverage of SSM within PHC systems	Improve planning, engagement, and team capacities to reflect and evaluate, fostered by peer exchanges	Conduct meetings to foster meaningful engagement, planning, feedback, and reflection
Inner setting	Adapt PHC structures to include a new group of “mentor of mentors” and create an implementation climate that promotes learning and a culture of data utilization	Create an organizational culture and climate that encourages learning and incentivizes feedback and adaptation between teams	Create an implementation climate that encourages learning and a culture of data utilization
Outer setting	Disseminate HIS guidance and procedures to emphasize SSM		Conduct public benchmarking of performance to incite friendly competition
Change mechanisms: Changes observed immediately after the start of SSM implementation			
	Improved motivation and skills at using interventions	Improved clarity and shared understandings of supportive supervision	Increased self-efficacy, improved perceptions of organization and teams, increased skill at using interventions
	Implementation climate that promotes innovation, learning, and improvement	Increased self-efficacy among supervisors, improved satisfaction with and self-identification within organization and teams	Implementation climate that drives and sustains quality improvement
	Greater accountability, planning, engagement, and execution of team tasks	Implementation climate that encourages quality improvement and learning	Increased confidence in SSM among implementers
Proximal outcomes: Results observed in the process evaluation			
	Improved use of data for health care worker performance	Reliably routine implementation of facilitative supervision	Improved use of time and resources based on data and strategic planning (i.e., implementing SSM where evidence shows it is needed)
	Improvement and problem solving at the point of care	Stronger linkages between levels of care (district, subdistrict, and CHPS zone)	Improved PHC worker adherence to clinical standards and guidelines
	Sustainable mechanisms for supervision, information sharing, and feedback between management and point of care	Improve management and performance of PHC at district, subdistrict, and CHPS zone levels	

Abbreviations: AHI, African Health Initiative; CBMP, capacity-building and mentorship program; CHPS, Community-based Health Planning and Services Program; FSV, facilitative supervision visits; HIS, health information system; PHC, primary health care; SSM, supportive supervision and mentoring.

### Phase 3: Sustainment

During their third, and current, phase, partnerships in Ethiopia, Ghana, and Mozambique articulate the contextual levels and factors that are key to the sustainability of the SSM interventions. These are salient insofar as they moderate the effectiveness of the strategy or fulfill essential preconditions for it to take effect. Similarities emerged across the 3 projects concerning moderators and preconditions. Factors that facilitated the success of SSM interventions are the enabling political environment, organizational culture, and collaborative links between government, academia, and civil society. In Ethiopia, the government has declared a “revolution” to move toward the universal use of data to drive quality improvement in health care and adopted a national strategy on the use of health data to link levels of care and guide SSM.^27^ The synergy between organizational culture, supportive policies, and the collaboration between research institutes and local government is key to sustaining quality PHC.

*We have made a great change in conducting implementation research… because we involve the district… and regional bureaus, we give them training, different kinds of training for them about implementation research about how to provide help out to prepare health profile, how to prepare different data visualization materials…. To use the data frequently, which are generated from the district.* —Academic research partner, Ethiopia

In the third phase, AHI partnerships articulate the contextual factors that are key to sustaining the SSM interventions.

In Ghana and Mozambique, stakeholders point out that the AHI partnerships engendered a culture within PHC networks that promotes innovation and learning. In Ghana, peer exchanges between district management teams were critical.

*One of the things that we did, where we have seen a lot of scale and services in CHPS implementation was when people went to study from others as changes came back and then also did extraordinary things because they want to be like them. The learning by doing, they are coming back. So that was very key. That mechanism for instance has been extraordinary for it improv[es] leadership at the district level.* —Ghana Health Service, national level, Ghana

Moreover, public health sector leaders from all AHI partnerships occupy roles that traverse policy and academic realms. Their reactions and reflections regarding SSM implementation illustrate how these individuals were uniquely placed to support the adaptation of the implementation strategy and sensitize local health systems to ensure uptake, sustainment, and fidelity to core intervention elements.

*When we started the project, I was in a leadership position at the Ministry of Health. I was engaged because I had the opportunity to discuss the priorities to define and to refine the research question. And from there, I was able to be part of the entire implementation process. There was a lot of engagement at the district and province level to sort of understand what the needs were and based on that we were able to define our specific project and approach.* —MOH official, national level, Mozambique

Despite the presence of these facilitating factors, SSM implementation in all countries faced barriers to long-term impact. Across projects, there were occasions when the organizational capacity to sustain SSM was suboptimal. Reports from Ghana highlighted persistent challenges related to logistics and transport as well as training other cadres of the health team. According to CHPS policy, subdistrict teams are required to monitor and supervise community health officers regularly, including budgeting for service delivery and general CHPS zone activities, periodically appraising community health officers’ technical skills, arranging equipment repair or replacement, and linking community health officers with peers for collaborative support. In Mozambique, a foremost impediment to sustaining the effective practice of SSM was health care worker retention.

*Frontline health care workers’ high turnover challenges the SSM implementation process since, very often, new colleagues need to be integrated and trained. Somehow this aspect may delay the implementation. Nevertheless, this situation was expected, and we just need to deal with it as we move forward.* —MOH official, Mozambique

In Ethiopia, AHI partners reported difficulty maintaining the fidelity to core elements of the CBMP, particularly for mentorship schedules and variability in the competency of mentors over time.

*In recent times, there is some slowing down of the momentum, and we are not seeing them (the mentors) coming that much. But they were doing it consistently before.* —Health facility director, Ethiopia

Even though CBMP mentors are expected to have detailed technical knowledge in different HIS areas, the need to deploy mentors from multiple disciplines to meet coverage needs as the program expanded resulted in salient skill gaps among CBMP mentors.

*I put their (the mentors) level of competency at the middle, since some simply fill checklist and go without discussing about the identified gaps or showing us ways to address them.* —District-level maternal, newborn, and child health focal person, Ethiopia

*The main gap observed was that some mentors were unable to support on DHIS-2 for they were not trained. The issue was reflected during our review meeting. To solve this gap, we gave them basic training on DHIS-2.* **—**AAU academic partner/health information, Ethiopia

Table 4 distills the similarities and differences of the SSM interventions conducted by the respective projects in Ethiopia, Ghana, and Mozambique.

**TABLE 4.** Comparison of the AHI SSM Implementation Strategies Carried Out in Ethiopia, Ghana, and Mozambique

**Similarities**	**Differences**
Made process improvements, usually at the management level, to address weak institutional capacities for planning, reflecting, and executing SSM at the district levels.	In Ethiopia and Mozambique, capacity building focused on technical skill building.
	In Ghana, this aimed at bolstering individuals’ understanding of supervision and their roles as mentors.
Promoted better learning climate and circulation of goals and feedback in delivery settings.	The Ethiopia team created a firmer structure and guidance for mentorship within national health information system policy, revised health worker roles to better emphasize mentorship and coaching, and established multidisciplinary mentorship teams to operationalize it.
	Mozambique used transparent benchmarking of quality improvement and peer exchanges between PHC teams.
	Ghana blended approaches by creating a subdistrict-level supervisor cadre to support community health workers and facilitate peer exchanges.
Enhanced readiness for implementation with better tools and catalytic funds.	In Ethiopia and Ghana, expansion of SSM was achieved through training cascades that link district management to points of care.
	In Mozambique, SSM implementation was based on evidence of the need to promote SSM quality where it is most required.
Increased frontline worker and supervisor understanding of SSM and clinical and nonclinical skills (e.g., data interpretation and utilization).	In Ghana and Mozambique, hindrances were mainly insufficient financial and material resources to ensure adequate implementation.
	Barriers to sustainment in Ethiopia related to lapses in fidelity to the core elements of the CBMP as the intervention grows.
Introduced a new cadre and process strategies to extend SSM to peripheral levels.	
Facilitated use of data to enhance quality and soundness of implementation both in terms of “real-time” decision making and long-term performance improvement.	
Fostered an enabling policy environment, collaborated between local health systems and academia, and embedded policy makers in research and policy domains.	
Held peer exchanges across jurisdictions to support the adoption and quality of SSM.	

Abbreviations: CBMP, capacity-building and mentorship program; PHC, primary health care; SSM, supportive supervision and mentoring.

## DISCUSSION

Global evidence and experience about implementing and sustaining SSM can be challenging to review. The imperative to customize to the needs and contexts of intervention settings has constrained the ability to compare successes and setbacks and generate generalizable knowledge or recommendations. In addition, supervision and mentoring efforts are often conceived as components of a complex intervention or as strategies for achieving high coverage of quality health services, resulting in the tendency to underexplore and underreport their operation and improvement in publications and reports.^18^ In recent decades, supportive supervision has become widely recognized as an important component of strengthening health service delivery and improving quality.^28^ Advocates recommend supervision as a strategy to reinforce health worker skills training; monitor and evaluate the availability of essential commodities, staff, and services; document and improve quality of care; and sustain the motivation of facility-based and community health workers. Lack of adequate supportive supervision is frequently cited as a barrier to implementing high-quality services.^29^^,^^30^ Innovations such as recommended schedules, checklists, and mHealth technologies have been devised to help standardize supervision encounters.^31^^,^^32^ The SSM strategies described in these 3 settings also point out the importance of ensuring a motivating and encouraging relationship between supervisors and their charges, which may be as valuable as the supervision framework and frequency.^33^^,^^34^ In cases where supervision initiatives have been rigorously evaluated on their own, evidence indicates that supervision can have large effects on health worker performance. More commonly, their impact has been more moderate, and they may be most effective when combined with other strategies.^35^ Even less is known about the potential effect of mentoring. Moreover, supervision and mentoring can support a range of clinical and nonclinical health system activities and a language for comparing approaches across these applications has yet to emerge.^18^

SSM strategies described in these 3 settings highlight the importance of ensuring a motivating and encouraging relationship between supervisors and their charges, which may be as valuable as the supervision framework and frequency.

Finally, even in an embedded science environment, the needs and priorities of health officials may not align well with typical research design approaches.^36^ The experiences of AHI partnerships described in this article are no exception to this. In Mozambique, the intervention design focused specifically on newborn survival. However, feedback from implementation stakeholders indicated that a more holistic PHC focus was needed, which resulted in the project adopting a more inclusive intervention that, in addition to focusing on newborn mortality reduction, also addressed a wider range of PHC issues. In Ghana, AHI researchers have strategically opted against use of the cluster-randomized controlled trial even though it is often touted as the “gold standard” evaluation design. Instead, these investigators prioritize relevance to policy makers and implementation leaders that believe that units of observation in evaluation research should conform to units of programmatic decision making. Accordingly, the AHI team in Ghana chose to use a plausibility evaluation design and conduct a quasi-experiment whose unit of analysis is the district.^37^ In the design of this study, the SSM working group strove to overcome these challenges and generate usable knowledge about local context, processes, and outcomes, and, to that effect, codesigned and carried out coordinated, theoretically sound, and analytically rigorous qualitative research that resulted in this article.

### Limitations

Even so, the cross-site work faces limitations. The highly situated nature of the 3 projects resulted in strategies that are comparable in some respects and distinct in others (Supplement Table). We struggled with finding a common language to describe the shared element and finally selected SSM as the focus of this analysis. The theoretical frameworks applied to this cross-national comparative study may not have been consciously applied with equal commitment across the sites. However, the working group was able to design data collection and analysis approaches to surface key domains from each of the models across all 3 settings.

Each of the sites operates on a slightly different timeline and strategies were initiated, assessed, and modified iteratively. The cross-national assessment was timed to coincide roughly with each project’s midpoint in terms of the multiyear funding cycle. But the interventions and implementation strategies were developed and implemented at different points in time, and it was not possible to assess the final outcomes or impact in this analysis. Nonetheless, the ability to compare experiences and perceptions of key actors in the development and initial deployment of SSM strategies has value of its own. In fact, a systematic review of the EPIS framework specifically called for researchers to apply the model more deliberately in the exploration and preparation phases of work.^22^ All 3 project teams demonstrated a focus on sustainment from the initial stages and were able to identify challenges that could improve the long-term success of their SSM efforts. However, also they noted that a variety of factors—most of which emanated from the encompassing health systems context—undermined sustainment. Most of these were acknowledged during the exploration and preparation phase (e.g., inadequate resources for SSM, workforce shortages, and logistics problems).

Despite the inclusive partnership model, the barriers to the sustainment of effective SSM suggests a need for a more integrated policy and multisectoral framework for diagnosing root cases of problems and engineering solutions that combine interventions at the point-of-care and management-level elements of the strategy. These elements tended to work well, with overarching improvements to the systems context that can be addressed by partnering with organizations that manage the supply chain, public sector authorities that regulate transfer and turnover of health care staff, and technical training institutions that can help mainstream in-service trainings to address mentor skill gaps beyond that which AHI partners did alone. The AHI partnership may be able to revisit this comparison when the projects have completed their final evaluation steps. However, at this point, we can glean lessons about early stages and implementation research design.

## CONCLUSIONS AND RECOMMENDATIONS

Each project team developed recommendations for improving SSM that may offer lessons for other contexts. Building in a participatory and shared approach such as coaching and mentoring to the workings of primary health care systems and management processes was emphasized by stakeholders of all projects, all of whom noted the importance of allocating adequate personnel and logistics, resources, and customizing the practice so that it improves knowledge and skills—both technical and motivational. While supervision checklists can be valuable tools, they should not constrain the supervision encounter. Otherwise, the opportunity for interactive and innovative problem solving can be missed. Incorporating experience and perspective from peers enhanced the acceptability and perceived effectiveness of SSM approaches, as did concomitant activities to encourage and strengthen data use for decision making. Through the phases of developing and adapting SSM interventions, all AHI teams benefited from their overarching embedded research strategies that promoted learning by doing and incorporated user reactions into intervention refinements.

We took effort to arrive at a common language to describe SSM strategies undertaken in the 3 settings. But doing so allowed us to collect comparable data and analyze the strategies in a consistent way while preserving the importance of informing local programs. We found that using a combination of implementation science models and frameworks led to comparative insights into the developmental stages of SSM strategies and to potential for longer-term successes. This suite of theoretical tools gave us the opportunity to look in detail at the 3 contexts and guided our comparisons across them without being overly specified or too burdensome. Working together on this assessment enhanced the ability of investigators and implementers to apply these models and frameworks across their work more broadly. Our experience may encourage other investigators to examine other SSM initiatives in a similar way. It also draws additional attention to the challenge of assembling generalizable knowledge about implementation across a field where context-specific learning is paramount.

**AHI Partnership Collaborative for Supportive Supervision and Mentoring Authors:** Colin Baynes (Department of Global Health, University of Washington, Seattle, WA, USA); Hiwot Belay (Ethiopia Data Use Partnership, John Snow International, Addis Ababa, Ethiopia); Lisa Hirschhorn (Feinberg School of Medicine, Northwestern University, Chicago, IL, USA); Quinhas Fernandes (Department of Global Health, University of Washington, Seattle, WA, USA; Ministry of Health, Maputo, Mozambique); Stephen Patrick Kachur (Mailman School of Public Health, Columbia University, New York, USA); Hibret Tilahun (Ethiopia Data Use Partnership, John Snow International, Addis Ababa, Ethiopia); Biruk Abate (Ministry of Health, Addis Ababa, Ethiopia); Mohammed Ahmed (Ministry of Health, Addis Ababa, Ethiopia); Ayaga A. Bawah (University of Ghana, Accra, Ghana); J. Koku Awoonor-Williams (Ghana Health Service, Accra, Ghana); Adriana Biney (University of Ghana, Accra, Ghana); Mawuli Kushitor (University of Ghana, Accra, Ghana); Mallory Sheff (Mailman School of Public Health, Columbia University, New York, USA); Nicholas Kanlisi (Mailman School of Public Health, Columbia University, New York, USA); Robert Alirigia (Ghana Health Service, Accra, Ghana); Pearl E. Kyei (University of Ghana, Accra, Ghana); James Phillips (Mailman School of Public Health, Columbia University, New York, USA); Kenneth Sherr (Department of Global Health, University of Washington, Seattle, WA, USA); Isaías Ramiro (Health Alliance International Maputo, Mozambique); Sarah Gimbel (Department of Global Health, University of Washington, Seattle, WA, USA; Department of Child, Family, and Population Health Nursing, University of Washington, Seattle, WA, USA), Dorlim Uetela (Department of Global Health, University of Washington, Seattle, WA, USA; Instituto Nacional de Saúde, Maputo, Mozambique); Orvalho Augusto (Department of Global Health, University of Washington, Seattle, WA, USA; Eduardo Mondlane University, Maputo, Mozambique); Celso Inguane (Department of Global Health, University of Washington, Seattle, WA, USA); Sergio Chicumbe (Instituto Nacional de Saúde, Maputo, Mozambique); and Eusebio Chaquisse (National Directorate of Public Health, Ministry of Health, Maputo, Mozambique).

## Supplementary Material

GHSP-D-21-00667-supplement.pdf

## Translation

**Em Português**

**Melhorando a Qualidade dos Cuidados Primários Através da Supervisão de Apoio e Mentoria: Lições da Iniciativa de Saúde Africana na Etiópia, Gana e Moçambique**

**Principais Conclusōes**

■ Os benefícios surgiram da supervisão de apoio e estratégias de mentoria que abordaram os factores multiníveis que operam em diferentes camadas de contexto em torno do ponto de atendimento, incluindo atitudes no nível do trabalhador e barreiras individuais para a melhoria do desempenho, até que ponto a aprendizagem e a adaptação são aceites e incentivados em ambiente mais amplo e estruturas e recursos colaborativos ao nível distrital disponíveis aos gestores e equipes para monitorar o desempenho.

■ Intercâmbios entre pares de equipes de cuidados de saúde primários (CSP), parcerias acadêmico-implementador e pesquisa de implementação incorporada foram fundamentais para o sucesso da supervisão de apoio e mentoria. Sua enfase na partilha de conhecimento e incorporação de suporte técnico e atividades de capacitação focadas no uso de dados para tomada de decisões e o aproveitamento das oportunidades do aprendizado motivacional em ambientes de prática rotineira.

**Principais Implicações**

■ As partes interessadas que têm influência sobre as práticas directas, processos e contexto mais amplo de supervisão de apoio e mentoria para os cuidados primários de qualidade devem estar engajados no diagnóstico de problemas de qualidade e no desenvolvimento coordenado de soluções holísticas que abordem os determinantes aos diferentes níveis de como a qualidade do cuidado é gerada e mantida.

■ Os gestores e parceiros de implementação poderão considerar formas de incorporar o aprendizado e a colaboração entre as equipes de CPS em todos os níveis e promover parcerias multissectoriais para sustentar a motivação e construir capacidade para uma melhor supervisão de apoio, mentoria e a melhoria do desempenho.

**RESUMO**

**Introduction:** A supervisão de apoio e mentoria (SAM) são cruciais para a qualidade e eficácia dos cuidados primários. No entanto, há pouca clareza sobre como projetar e implementar o SAM e torná-lo sistemas sustentáveis de cuidados de saúde primários (CPS). Os 3 projetos de parceria da Iniciativa Africana de Saúde (AHI) introduziram estratégias para fazer isso na Etiópia, Gana e Moçambique. Descrevemos: (1) como cada parceria adaptou as estratégias de implementação da SAM, (2) as dinâmicas de implementação e mudança que se seguiram após a intervenção nos sistemas de CSP e (3) percepções sobre a sustentabilidade da SAM como pilar dos CSP.

**Métodos:** Pesquisadores de cada projecto escreveram colaborativamente um protocolo entre os países com base nesses objetivos. Para isso, eles adaptaram as estruturas da ciência de implementação: o modelo de Exploração, Preparação, Implementação e Sustentação e Estrutura Consolidada para Pesquisa de Implementação por meio de um processo qualitativo de redução temática. Isso resultou em linhas de investigação harmonizadas sobre a concepção, implementação e sustentabilidade potencial da estratégia de SAM de cada projecto. Entrevistas aprofundadas e discussões de grupos focais foram realizadas com as partes interessadas dos sistemas de CSP em cada país e as análises temáticas se seguiram.

**Resultados:** Entre os projetos, as estratégias de SAM adquiriram vários componentes para abordar os determinantes individuais, de sistemas e relacionados a processos. Os benefícios surgiram dos esforços que abordaram as atitudes e barreiras do trabalhador, promoveram um ambiente de aprendizagem mais amplo e melhoraram as estruturas e ferramentas de colaboração para monitorar o desempenho. Intercâmbios entre pares e pesquisa de implementação incorporada foram fundamentais para a eficácia percebida das estratégias de SAM.

**Discussão:** Apesar das diferenças na abordagem à implementação do SAM, existem ingredientes cruciais comuns nas estratégias do SAM dos 3 projetos parceiros da AHI, dos quais surgem lições importantes: (1) posicionar as oportunidades de aprendizagem e adaptação dentro do funcionamento de rotina dos sistemas de CSP, facilitação e suporte técnico para reflectir e utilizar novos conhecimentos; (2) colaboração multissetorial, particularmente com organizações acadêmicas; e (3) capacitar os tomadores de decisão da CSP e as equipes de implementação para mudanças baseadas em evidências.

**Peer Reviewed**
