# Supplementary material for: Improving Primary Care Quality Through Supportive Supervision and Mentoring: Lessons From the African Health Initiative in Ethiopia, Ghana, and Mozambique
Source: Glob Health Sci Pract. 2022 Sep 15;10(Suppl 1):e2100667. doi: 10.9745/GHSP-D-21-00667 (PMC9476486; doi:10.9745/GHSP-D-21-00667)
Supplement: GHSP-D-21-00667-supplement.pdf [file GHSP-D-21-00667-supplement.pdf]

Supplement to: AHI Partnership Collaborative for Supportive Supervision and Mentoring. Improving primary care quality through supportive supervision and mentoring: lessons from the African Health Initiative in Ethiopia, Ghana, and Mozambique. *Glob Health Sci Pract*. 2022;10(Suppl 1): e2100667. https://doi.org/10.9745/GHSP-D-21-00667

Supplement Table. Causal Pathway Model Terms and Definitions

Term	Definition
Implementation strategy	Set of activities identified based on assessment of program stakeholders, contexts, and likely barriers and facilitators that aims to trigger systems changes and achieve better primary health care worker performance.
Determinants	Factors that enable or hinder the implementation strategy for eliciting the desired effect (i.e., barriers and facilitators).
Preconditions	Factors or circumstances necessary for an implementation strategy to trigger desired systems changes and achieve desired outcomes.
Action targets	The level of the health system or context at which determinants operate.
Outer setting	The economic, political, policy, social, and cultural context within which the implementing organization resides.
Inner setting	The entities that comprise the implementing organization, including its structural characteristics, communication networks, culture, climate, and readiness to implement.
Individual	Characteristics of the individual people that are members of the implementing organization (e.g., their skills and competencies, self-efficacy, and self-identification within their organization).
Process	The ways in which members of the implementing organization plan, engage, execute, reflect, and evaluate to carry out the implementation strategy.
Mediator	Factors or processes that account for the relationship between the implementation strategy and the implementation outcome (i.e., how an implementation strategy has an effect).
Moderators	Factors that increase or decrease the influence and effectiveness of an implementation strategy.
Change mechanisms	The event through which an implementation strategy operates to affect desired outcomes.
Proximal outcomes	The product of the implementation strategy that is realized because of its specific change mechanism; the immediate, observable outcome in the pathway.
